# Lichen Planopilaris As Part of Multiple Autoimmune Syndrome: A Case Report of New Association

**DOI:** 10.7759/cureus.52892

**Published:** 2024-01-24

**Authors:** Heba R Hamad, Shoroq Alamin, Moteb A Alotaibi

**Affiliations:** 1 College of Medicine, Sulaiman Al Rajhi University, Al-Bukayriyah, SAU; 2 Medicine, Unaizah College of Medicine and Medical Sciences, Qassim University, Unaizah, SAU

**Keywords:** multiple autoimmune syndrome (mas), hypothyrodism, (ibd) inflammatory bowel disease, lichen planopilaris (lpp), cicatricial alopecia

## Abstract

Lichen planopilaris (LPP) is an uncommon inflammatory scalp condition. Its typical clinical presentation includes scaly, erythematous plaques resulting in irreversible alopecia. In this study, we report a female in her late 30s with hypothyroidism and Crohn’s disease. She presented with gradual, localized hair loss that had been ongoing for the past four months. A thorough physical examination, and complemented by dermoscopic evaluation, confirmed the diagnosis of LPP. Individuals who have an autoimmune disease (AID) have a heightened propensity to develop additional AID. The coexistence of three or more AIDs falls under the definition of multiple autoimmune syndrome (MAS). This is the first case, to the best of our knowledge, of LPP being associated with MAS.

## Introduction

Lichen planopilaris (LPP) is an uncommon inflammatory scalp disorder clinically characterized by perifollicular erythema, follicular hyperkeratosis, and cicatricial alopecia [1]. The incidence of LPP varied from 1.15% to 7.59% and the common age of onset is between 25 and 70 years [1]. LPP is considered a follicular form of lichen planus based upon shared pathological features and the frequent coexistence of clinical findings of these disorders [2]. The autoimmune process in LPP refers to the hair-specific disorder, where active T lymphocytes target follicular antigens, leading to inflammation and destruction of hair follicles in the scalp [2]. The autoimmune nature of LPP has been further supported by studies exploring the associations between it and other cutaneous autoimmune diseases, like psoriasis and vitiligo [3]. These disorders share a common mechanism of T cell-mediated immune responses [3]. In this unique case, the patient presented with LPP associated with hypothyroidism, a well-established combination that has been observed before in the literature. However, the interesting new development was the presence of Crohn's disease, which suggests a more complex autoimmune involvement known as multiple autoimmune syndromes (MAS).

## Case presentation

**Figure 1:**
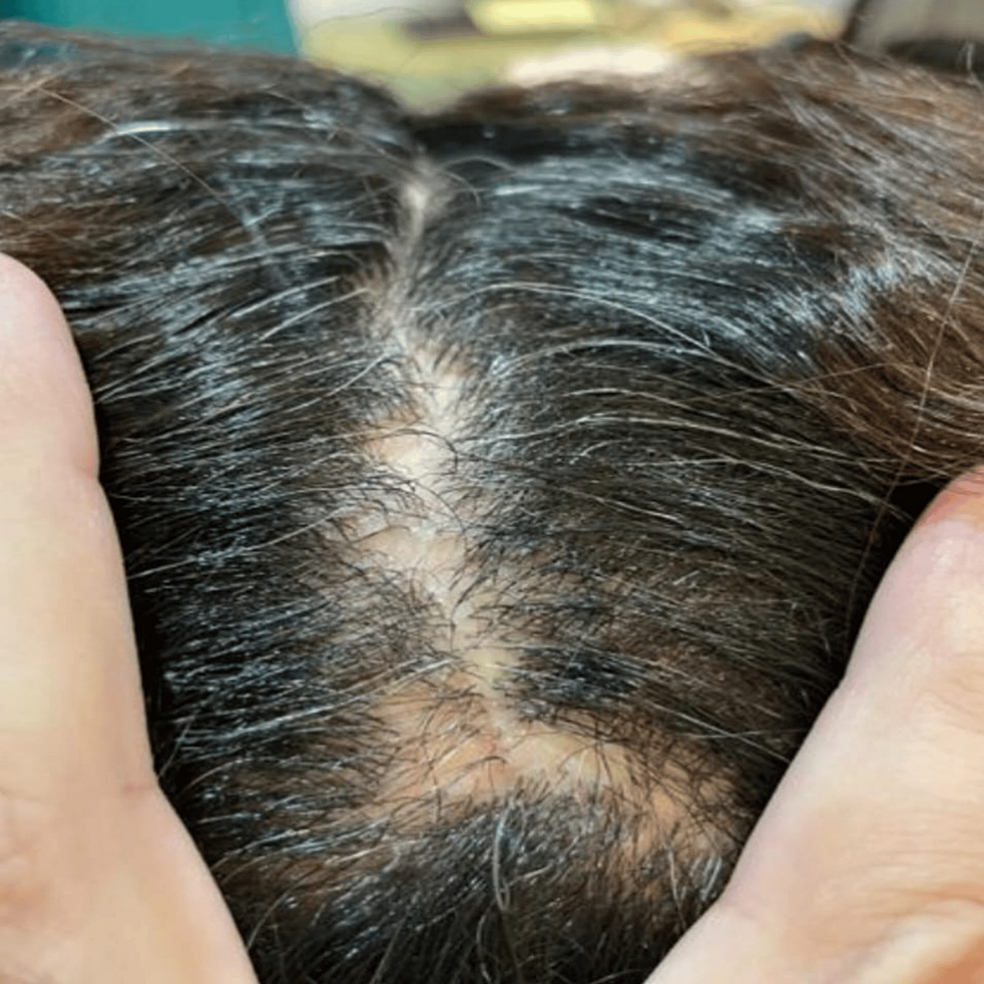
Multiple alopecic patches on the vertex area around the middle hairline.

A female in her late 30s presented with localized hair loss for the past four months. It started as a gradual hair loss in the middle hairline, then stopped a few weeks ago. There were no itching, tenderness, or bothersome scales. She has no current or past history of skin, mucous membranes, or nail problems. Physical examination revealed multiple alopetic patches on the vertex area around the middle hairline (Figure 1). Skin, mucous membranes, and nails were normal. Dermoscopy examination showed a complete absence of follicular openings, absence of vellus hairs, pale-white areas in the centers, and perifollicular scales, mainly at the lesions' margins. A skin biopsy procedure was planned, but the patient refused as she was afraid of any invasive procedure.

Upon further inquiry, she was diagnosed with Crohn's disease five years ago, and she has received a course of corticosteroids then the condition was maintained on azathioprine 100 mg daily till now. She has hypothyroidism that was discovered two years ago and maintained on levothyroxine 150 mcg daily. One year ago, she developed recurrent episodes of unilateral facial pain of a burning nature that was diagnosed later as trigeminal neuralgia. For her neuralgia, she was prescribed topiramate 50 mg daily. Recently, she started to experience episodes of hyperesthesia on her upper limbs without any motor problem, but no diagnoses have been reached till now. Her recent laboratory investigations including complete blood count and comprehensive metabolic panel were within normal limits. Given the typical clinical features of her hair loss, the diagnosis of lichen planopilaris was made. Moreover, her medical history was suggestive of multiple autoimmune syndromes. Consultation with relevant medical specialties was done to reach a suitable treatment plan to address all the current problems.

## Discussion

Lichen planopilaris (LPP) is an uncommon, scarring, lymphocyte-mediated alopecia that is presumed to have an autoimmune pathogenesis and may be associated with other autoimmune conditions [1]. LPP has been described as the most common primary cicatricial alopecia. It predominates in females, typically in the age range of 40-60 years. Approximately half of the patients with lichen planus lesions may exhibit typical manifestations that involve the skin, mucous membranes, or nails [2]. The typical clinical presentation of LPP is scaly, erythematous plaques of alopecia, occasionally accompanied by ulceration and atrophy, as well as perifollicular erythema, follicular hyperkeratosis, and irreversible alopecia [1]. The pathophysiology of LPP is based on a T cell-mediated inflammation that targets the stem cell area within the hair follicles, also known as the infundibulo-isthmic or bulge region, leading to irreversible alopecia and follicular scarring [1]. T cells, particularly T helper cells, such as Th1, Th9, Th17, Th22, and cytotoxic T lymphocytes, play a significant role in the excessive immune response to keratinocyte cell death [3]. The JAK/STAT pathway has also been shown to contribute to the development of this condition [4].

Thyroid disease is one of the most common comorbidities associated with LPP and has been extensively studied in the literature. Old studies have reported a positive association between LPP and thyroid disease, especially hypothyroidism and autoimmune thyroiditis [5,6]. Even the latest studies agree with these findings, such as the case-control study conducted using the all-US database. The authors proposed that LPP and hypothyroidism may share a common pathogenesis involving dysregulated T cell/cytokine pathways, as both conditions are T lymphocyte-mediated processes [7]. However, the relationship between LPP and other autoimmune diseases besides thyroid disease remains unclear, as different studies have reported contradictory results. Two studies, one by López-Jornet et al. and another by Brankov et al. found no significant association between LPP and autoimmune diseases, except for thyroid disease [1,8]. More recent studies revealed a higher prevalence of LPP and autoimmune disease, especially hypothyroidism, inflammatory bowel disease (IBD), rheumatoid arthritis, and systemic lupus erythematosus [7,9-11]. Two of these conditions, hypothyroidism and IBD, were also found in the present case, with the main complaint of LPP. These three disorders presenting with each other have contributed to multiple autoimmune syndromes (MAS).

Multiple autoimmune syndrome (MAS) is the paradigm of polyautoimmunity. Emphasizing this concept, a study has shown that approximately 25% of individuals with an autoimmune disease are more likely to develop additional autoimmune disorders later in life [12]. Clinically, MAS is defined as the concurrence of three or more autoimmune diseases (AIDs) in one person [13]. In the present case, they were lichen planopilaris, hypothyroidism, and Crohn's disease. The world prevalence is estimated at 3-9.4% with a well-recognized increasing frequency. Based on the prevalence of AIDs co-occurrence with each other, MAS has been classified into three types. However, the coexistence of AIDs is expanding beyond these groups. Multifactorial pathological mechanisms cause the development of MAS. In a 2023 study, Fidalgo et al. proposed the genetic background hypothesis, highlighting the HLA-DRB1*03 gene as a significant risk factor [14]. In patients with MAS, it is common to have at least one dermatological condition, usually vitiligo or alopecia areata. Other autoimmune diseases that have been documented in the literature include bullous pemphigoid, pemphigus, psoriasis, dermatitis herpetiformis, lupus erythematosus/lichen planus-overlap syndrome, scleroderma, cicatricial pemphigoid, and cutaneous and oral lichen planus [8,15-20].

## Conclusions

Our extensive literature search shows that this is a novel, first-time reported case of LPP being associated with MAS. The presence of one autoimmune disease should alert healthcare professionals to the possibility of developing another autoimmune disease in the MAS spectrum. Providing the chance for early diagnosis and proper management. Further investigation is needed to explore the pathogenesis resemblance of these diseases that could influence the choice of immunotherapy.
